# A statistical framework to evaluate virtual screening

**DOI:** 10.1186/1471-2105-10-225

**Published:** 2009-07-20

**Authors:** Wei Zhao, Kirk E Hevener, Stephen W White, Richard E Lee, James M Boyett

**Affiliations:** 1 Department of Biostatistics, St Jude Children's Research Hospital, Memphis, TN, USA; 2 Department of Pharmaceutical Sciences, University of Tennessee Health Science Center, Memphis, TN, USA; 3 Department of Structural Biology, St Jude Children's Research Hospital, Memphis, TN, USA; 4 Department of Molecular Sciences, University of Tennessee Health Science Center, Memphis, TN, USA

## Abstract

**Background:**

Receiver operating characteristic (ROC) curve is widely used to evaluate virtual screening (VS) studies. However, the method fails to address the "early recognition" problem specific to VS. Although many other metrics, such as RIE, BEDROC, and pROC that emphasize "early recognition" have been proposed, there are no rigorous statistical guidelines for determining the thresholds and performing significance tests. Also no comparisons have been made between these metrics under a statistical framework to better understand their performances.

**Results:**

We have proposed a statistical framework to evaluate VS studies by which the threshold to determine whether a ranking method is better than random ranking can be derived by bootstrap simulations and 2 ranking methods can be compared by permutation test. We found that different metrics emphasize "early recognition" differently. BEDROC and RIE are 2 statistically equivalent metrics. Our newly proposed metric SLR is superior to pROC. Through extensive simulations, we observed a "seesaw effect" – overemphasizing early recognition reduces the statistical power of a metric to detect true early recognitions.

**Conclusion:**

The statistical framework developed and tested by us is applicable to any other metric as well, even if their exact distribution is unknown. Under this framework, a threshold can be easily selected according to a pre-specified type I error rate and statistical comparisons between 2 ranking methods becomes possible. The theoretical null distribution of SLR metric is available so that the threshold of SLR can be exactly determined without resorting to bootstrap simulations, which makes it easy to use in practical virtual screening studies.

## Background

Structure-based Virtual screening (VS), the process of docking three-dimensional (3D) models of drug-like compounds into 3D models of potential drug receptors, has become an integral part of the drug discovery process in recent years [1-5]. VS is the computational analog of biological screening and is used to score, rank, and/or filter a set of structures by using one or more computational procedures. Such computational methods are faster and more cost-effective than physically testing several thousand potential drugs in chemical or cell-based assays, which has been the norm in the pharmaceutical industry for decades. Because *in silico *screens are faster and less expensive than those performed by high-throughput screening (HTS) methods, VS methods can effectively limit the number of compounds to be evaluated by HTS to a subset of molecules that are more likely to yield "hits" when screened. It has also been shown that the "hits" from VS when compared to HTS are often diverse and independent, suggesting that VS and HTS might be considered complementary methods [6,7]. Brenk et. al [7] went on to show that VS has the ability to identify "hit" compounds that can potentially be missed by traditional HTS methods, but can still be developed into acceptable lead compounds.

Structure-based VS involves the docking of candidate ligands into a protein target and estimating the affinity of ligand receptor binding through a scoring function. A key requirement for the success of VS is the ability of the combination of a docking method and scoring function to rank actives early in a large set of compounds [3,6,7]; we refer to such combinations as *ranking method *hereafter. Many metrics, AU-ROC, RIE, BEDROC, pROC etc., are currently used to evaluate the performance of ranking methods in VS studies [8-17]. However, no docking validation paper to date has utilized significance testing and rigorous statistical analysis in evaluating virtual screening studies, although this has been recommended by Cole et al. [12]. We are the first to propose such a statistical framework to compare virtual screening studies and the method has been successfully used in [18] to compare difference ranking methods.

Receiver operating characteristic (ROC) curves have been widely used to evaluate VS methods [16,17,19-22]. An ROC curve is a plot of true-positive rates versus false-positive rates for all compounds. The area under the ROC (AU-ROC) curve is the probability of active compounds being ranked earlier than decoy compounds. AU-ROC is a well-established statistical method that is also widely used in many other disciplines [23-25]. Several parametric and nonparametric statistical methods have been developed for significance testing [26-32].

Although widely used, AU-ROC is not a good metric to address the "early recognition" problem specific to VS, as pointed out by Truchon and Bayly [16]. Since AU-ROC is equivalent to a simple average of the ranks of the actives, the good performance of "early recognitions" is offset quickly by "late recognitions". Let *n *be the number of actives and *N *be the total number of compounds, AU-ROC is approximately normal distributed, with mean  and variance . AU-ROC defined in equation (1) is linearly related to the rank sum of actives, which is also called Mann-Whitney U test. *r*_*i *_is the rank of the *i*th active.

(1)

**Figure 1:**
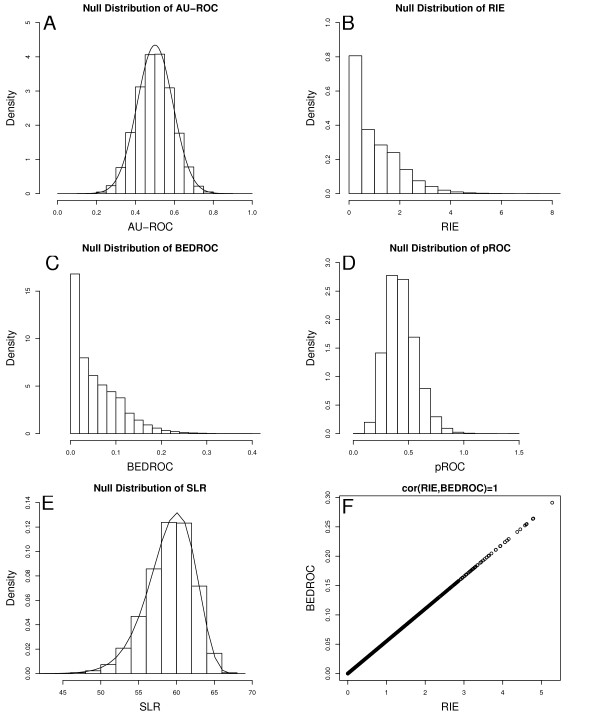
**Empirical distributions of ranking scores under the null hypothesis that 10 actives are uniformly distributed, 1000 compounds in total**. (A) AU-ROC, smooth lines indicate normal distribution with mean  = 0.5005 and variance  = 0.0084; (B) RIE; (C) BEDROC; (D) pROC; (E) SLR, 10 × log(1000)-SLR is Gamma(10, 1) distributed; and (F) the perfect linear relationship between RIE and BEDROC.

Figure 1(A) shows how close the normal approximation is to the empirical distribution derived from bootstrap.

Truchon and Bayly [16] have discussed several methods to address the problem of "early recognition". They have shown that the exponential weighting schemes, BEDROC and robust initial enhancement (RIE), provide good "early recognition" of actives. By changing the tuning parameter, *α*, users can control the earliness of "early recognition" to test whether a ranking method is useful in the context of VS. BEDROC is bounded by interval [0, 1] and can be interpreted as the probability that an active is ranked before a randomly selected compound exponentially distributed with parameter *α*, only when *α *<< 1. RIE, developed by Sheridan et al [14], used an exponential weighting scheme, that places heavier weight to "early recognized" actives.

(2)

where *x*_*i *_=  is the relative rank of the *i*th active and α is a tuning parameter. BEDROC is derived from RIE and it is bounded by [0, 1]. BEDROC has a linear relationship with RIE,

(3)

Although RIE and BEDROC produce different values, their distributions are identical up to a scale and a translation factor and their correlation is 1, as shown in Figure 1B, C and 1F.

Logarithmic transformation shifts the emphasis from "late recognition" to "early recognitions". Instead of working on the ranks of the actives, Clark and Clark [15] proposed a new metric, pROC, on basis of the negative logarithmic transformation of false positive rates, *θ*. When the false positive rate is zero, Clark and Clark suggested a zero-point continuity correction be made by replacing zero with 1/N.

(4)

It is heuristic to compare different ranking methods using all the aforementioned metrics, but whether a ranking method is better than random ranking or whether 2 ranking methods are truly different is yet unclear. In this study, we have analyzed and compared the various metrics used in VS by extensive computer simulations. We have laid down a statistical framework that can rigorously determine thresholds and compare different ranking methods by using AU-ROC, BEDROC, RIE, pROC, or other metrics. We present a bootstrap method to generate null distribution for any metric and then discuss how a meaningful threshold can be selected. We also introduce a permutation test that allows comparisons between 2 ranking methods and give the criterion under which their difference can be claimed as being statistically significant.

## Methods

### Bootstrap method to generate null distributions

For any ranking method, it needs to be determined whether a score, whether it is from AU-ROC, BEDROC, RIE, or pROC, is better than random ranking. Without knowing the distribution of the metric under the random ranking assumption, it is impossible to select an appropriate threshold. An *ad hoc *threshold determined through experience may help but is difficult to justify. For AU-ROC, a theoretical distribution can be derived by reasonable approximations; however, for other metrics, theoretic distributions are less obvious and difficult to derive. Bootstrap is an ideal method to derive theoretical distributions in such cases.

Bootstrap is a re-sampling method that has been widely used [33-35]. In statistics, null distribution is the distribution when the null hypothesis that the ranking method is no better than random ranking is true. Under this null hypothesis, ranks of the actives come from a uniform distribution, and the empirical distribution of a metric can be derived by repetitively drawing ranks from a uniform distribution over times. Current high-speed computers take less than a minute to process 1 million such repeats. Empirical distribution approaches true distribution when the number of repeats increases, and according to us 1 million repeats is sufficiently large to derive the empirical distribution of a metric. For example, in pROC, if we assume that there are 10 active compounds and 990 inactive compounds in a list, under the null hypothesis 10 actives are uniformly distributed so that their ranks can be drawn randomly with replacement from 1 to 1000. One pROC value is calculated in each repeat, and this process is repeated 1 million times to derive the empirical distribution of pROC. As a large pROC value indicates good early recognition, we can select a threshold large enough such that only a small percentage of pROC values are larger than the threshold. This small percentage is called the type I error rate, that is, the error rate of claiming that the ranking method is better than random ranking when it is actually truly random. The threshold is usually chosen to be the 95% percentile (5% type I error rate) of the null distribution; a more stringent threshold is often set at the 99% percentile (1% type I error rate) of the null distribution. On the other hand, we can also locate the observed pROC value on the null distribution. The probability that the null distribution is greater than the observed pROC is the *p *value. The ranking method is better than random ranking if the *p *value is less than 0.05. Because the ranks are drawn from a known uniform distribution, this bootstrap method is also called "parametric bootstrap".

Figure 1(A)–(D) shows the empirical null distributions of the AU-ROC, RIE, BEDROC, and pROC, respectively. All distributions are derived assuming there are *n *= 10 active compounds and a total of *N *= 1000 compounds, actives and in-actives included. In Figure 1(A), the smooth line on top of the empirical distribution of AU-ROC is the approximated theoretic distribution, which is a good approximation to the empirical distribution. Figure 1(B) and 1(C) show the empirical distribution of RIE and BEDROC at *α *= 20. These 2 distributions are identical in shape up to a scale and a translational factor; Figure 1(F) shows the perfect linear relationship between these 2 metrics. For the rest of this paper, we will assume RIE and BEDROC to be 1 method and will discuss BEDROC method only, as all the properties of BEDROC apply to RIE too.

**Figure 2:**
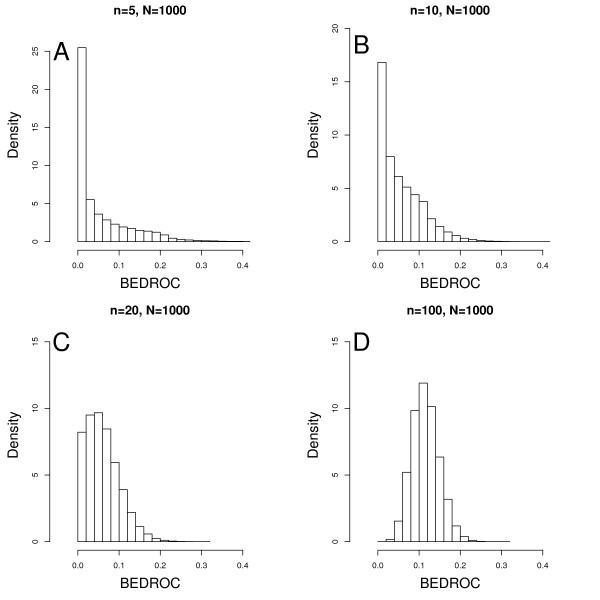
**Null distribution of BEDROC for different lists of compounds **(A) *n *= 5 and 95% threshold is 0.20; (B) *n *= 10 and 95% threshold is 0.16; (C) *n *= 20 and 95% threshold is 0.14; and (D) *n *= 100 and 95% threshold is 0.17.

Null distributions are not fixed and they change when the list of actives and inactives changes. For BEDROC, null distributions also change for different tuning parameters *α*. Figure 2 shows the distributions of BEDROC for different number of active compounds. It is obvious that the distribution becomes more centered when the number of actives increases so that the thresholds should also change accordingly. The central limit theorem predicts that the distribution will be approximately normal for large set of actives, but this is not true for a list of small number of actives. Although BEDROC is bounded by [0, 1], it does not always have a probability interpretation like AU-ROC does. Because 2 ranking methods may have different underlying distributions, they cannot be compared if ranks are derived from different sets of compounds or BEDROC has different tuning parameters.

### Sum of the log of ranks test (SLR)

In statistical theory, if *X *is uniformly distributed, -log(*X*) follows a *Gamma*(1,1) distribution [36]; if *X*_1_, *X*_2_, ..., *X*_*n *_are all independently uniformly distributed, then  follows a *Gamma*(*n*,1) distribution. Under null hypothesis,  follows a uniform distribution so that -log () is *Gamma*(1,1) distributed. On the basis of this theory, the theoretical distribution of the sum of the log of ranks statistic (SLR), , can be derived from Eq (5). Noted that a low SLR prefers "early recognition."

(5)

The threshold for the SLR statistic is easy to derive theoretically. Suppose *n *= 10 and *N *= 1000, the 95% percentile of *Gamma*(10,1) is 15.7. The 95% threshold for SLR is 10 log(1000) - 15.7 = 53.38. Thus a ranking method is deemed as statistically significantly better than random ranking if the observed SLR is less than 53.38. Figure 1(E) shows the empirical distribution of SLR under null hypothesis, with *n *= 10 active compounds and a total of *N *= 1000 compounds. The smooth line on top of the empirical distribution is the theoretical distribution derived from Equation 5.

Equation (4) can be rewritten as Eq (6) in terms of the ranks of actives. Except for the normalizing factor, the second parts of Eq (5) and Eq (6) are similar. The only difference is that SLR uses the rank itself but pROC subtracts the number of actives from the rank. Because of the subtraction, pROC place slightly more emphasize than SLR on "early recognition". As noted by Clark and Clark [15], pROC is unbounded when *r*_*i *_- *i *= 0, for which they recommended replacing the false positive by 1/N as a zero correction.

(6)

### Permutation test to compare two ranking scores

When 2 ranking methods that use the same compound list are both significantly better than random ranking, it is useful to find by using appropriate metrics whether 1 method is superior and whether the difference is statistically significant. Because the distribution of actives is no longer uniform for such comparisons, we have proposed a permutation test to compare 2 ranking scores.

The permutation test is a type of non-parametric test, the null hypothesis being that the 2 ranking methods are equivalent. Here we use the SLR method as an example to demonstrate the permutation test, but the technique is applicable for other metrics too. Under the equivalent assumption, the difference of SLR between 2 ranking methods, *x *and *y*, is zero, i.e. *SLR*_*x *_- *SLR*_*y *_= 0. Hereafter, we also use *x *and *y *to denote the set of ranks. Assuming that *x*_*i *_and *y*_*i *_are ranks of the *i*th active for the 2 ranking methods, the permuted ranks are given by  and , where by  and  are random samples from the pool of ranks (*x*, *y*). From this, the exact distribution of the difference between the 2 ranking methods can be calculated and the observed difference can be compared with the null distribution to determine whether the difference is significant. For example, let *x *= {55,2,4,16,150,1,3,7,215,744} and *y *= {27,65,47,595,158.5,200,22,440.5,223,40}be the ranks of 10 active compounds for 2 ranking methods from a list of 749 compounds. Our objective is to test whether *x *is significantly better than *y*. The observed difference is *SLR*_*x *_- *SLR*_*y *_= -17.45. A permuted sample becomes x* = {55,2,47,16,158.5,200,3,440.5,223,744} and y* = {27,65,4,595,150,1,22,7,215,40} and the new statistic of the permuted data is *SLR*_x* _- *SLR*_y* _= 6.98. Of the 2000 such permutations calculated, the observed test statistic is less than the permuted statistics 1922 times, which results in a p-value of 0.039. We therefore conclude that *x *is statistically significantly better than *y *at a type I error rate of 0.05.

## Results & Discussion

We use simulation studies to examine AU-ROC, SLR, BEDROC, and pROC in detail. In this section, we compare different metrics and investigate the conditions under which the metrics perform best. We also compare the statistical power of the metrics using the simulated data of ranking experiments.

### How early is early?

To compare the "earliness" that different metrics emphasize, we studied the average ranks of the earliest recognitions at 10%–100% of the total number of actives for the ranking methods deemed better than random ranking. The total number of compounds was *N *= 1000 and we increased the number of active compounds from *n *= 5 to *n *= 100 to examine the consistencies and pattern under different conditions. The null distribution of each metric is derived from 10,000 bootstraps and the threshold at 5% error rate is empirically determined. Since *α *= 20 for BEDROC is suggest by Truchon and Bayly [16], we use this parameter throughout our simulations.

Table 1 shows the proportion by which the average ranks of a metric are smaller than that of another metric. SLR, pROC and BEDROC all have better "early recognitions" than AU-ROC does for the first 30% and for most of the 40% of early recognized actives. SLR and pROC are better than AU-ROC for up to 50%; BEDROC starts performing worse than AU-ROC at 40% of the actives when *n *= 50 (data not shown) and this difference in performance becomes more obvious when *n *= 100. With *α *= 20, BEDROC clearly places heavier emphasis on the first 10% to 20% of actives than other metrics. Beyond 30%, both SLR and pROC have smaller average ranks than BEDROC. Overall, the performance of SLR and pROC are similar in rewarding early recognitions.

**Table 1:** Comparisons of the "earliness" of different metrics to detect early recognitions for different number of actives (*n *= 5, 10, 20, 100).

Metric 1	Metric 2	10%	20%	30%	40%	50%	60%	70%	80%	90%	100%
n = 5											

pROC	AU-ROC	0.80	0.80	0.70	0.70	0.70	0.56	0.42	0.42	0.42	0.32

BEDROC	AU-ROC	0.86	0.86	0.72	0.72	0.72	0.46	0.28	0.28	0.28	0.18

SLR	AU-ROC	0.79	0.79	0.72	0.72	0.72	0.58	0.43	0.43	0.43	0.32

SLR	pROC	0.52	0.52	0.52	0.52	0.52	0.52	0.51	0.51	0.51	0.52

SLR	BEDROC	0.44	0.44	0.47	0.47	0.47	0.61	0.66	0.66	0.66	0.67

pROC	BEDROC	0.46	0.46	0.48	0.48	0.48	0.60	0.65	0.65	0.65	0.67

n = 10											

pROC	AU-ROC	0.78	0.77	0.73	0.67	0.61	0.52	0.46	0.40	0.35	0.32

BEDROC	AU-ROC	0.81	0.87	0.76	0.62	0.49	0.38	0.30	0.23	0.20	0.18

SLR	AU-ROC	0.76	0.76	0.73	0.67	0.61	0.54	0.47	0.41	0.36	0.33

SLR	pROC	0.54	0.52	0.54	0.54	0.55	0.54	0.54	0.54	0.54	0.54

SLR	BEDROC	0.50	0.35	0.45	0.54	0.61	0.66	0.67	0.69	0.70	0.70

pROC	BEDROC	0.51	0.37	0.45	0.55	0.61	0.65	0.68	0.69	0.69	0.69

n = 20											

pROC	AU-ROC	0.77	0.75	0.71	0.66	0.58	0.52	0.45	0.39	0.35	0.33

BEDROC	AU-ROC	0.85	0.84	0.71	0.57	0.44	0.34	0.26	0.21	0.18	0.17

SLR	AU-ROC	0.77	0.75	0.71	0.66	0.60	0.54	0.47	0.41	0.36	0.34

SLR	pROC	0.51	0.52	0.53	0.53	0.53	0.53	0.53	0.53	0.53	0.53

SLR	BEDROC	0.40	0.38	0.50	0.58	0.65	0.69	0.71	0.72	0.72	0.72

pROC	BEDROC	0.41	0.38	0.48	0.58	0.63	0.66	0.69	0.70	0.70	0.70

n = 100											

pROC	AU-ROC	0.75	0.73	0.67	0.61	0.54	0.48	0.42	0.38	0.35	0.34

BEDROC	AU-ROC	0.87	0.79	0.62	0.47	0.36	0.28	0.22	0.19	0.17	0.16

SLR	AU-ROC	0.75	0.72	0.68	0.62	0.56	0.50	0.44	0.39	0.36	0.35

SLR	pROC	0.55	0.55	0.56	0.56	0.56	0.57	0.57	0.58	0.58	0.58

SLR	BEDROC	0.32	0.42	0.56	0.64	0.69	0.72	0.74	0.74	0.74	0.74

pROCa	BEDROC	0.34	0.43	0.56	0.63	0.68	0.70	0.72	0.73	0.72	0.72

The top early recognitions are represented as percentages.

As there is no solid criterion to judge earliness, one could potentially construct a new metric that emphasizes "early recognition" more than any existing metrics. However, whether the newly constructed metric is reasonable or not depends entirely on the user's discretion. Increasing *α *in BEDROC shifts the emphasis to in favor of earlier hits, and using weighting functions in SLR and pROC can achieve the same effect.

### Weighted SLR methods

The SLR method can be fine tuned to place more emphasis on early recognized active compounds by incorporating appropriate weighting functions. One weight function we consider is the powered arithmetic weights *w *= {*n*, *n *- 1, ...1}^*β*^, where *β *is the power. The weighted SLR metric is defined in Eq (7). Increasing the power increases the emphasis on "early recognition."

(7)

Tables 2 compare the "earliness" of wSLR at different *β *values with BEDROC for different number of actives. There is a general trend that wSLR improves against BEDROC in the top 10% list when *β *increases. When *β *is large, emphases are placed only on the several top active compounds in the list. This explains why BEDROC seemingly outperforms wSLR in the top 10% list for *n *= 100. When only looking at the top 1 active compound in the list [data not shown], it becomes much more clear that one can increase the weight of early recognitions by increasing the power of the weight function. It is worthwhile to mention here that the weight function is also another way to incorporate external information into the wSLR metric. For example, one can incorporate IC_50 _values of the actives to wSLR by applying the weight function to the ranks of their IC_50 _[18].

**Table 2:** Comparisons of the earliness of wSLR and BEDROC (*α *= 20) at different powers and number of actives, *β *= 0, 0.5, 1, 2, 5, 10, *n *= 5, 10, 20, 100.

*β*	10%	20%	30%	40%	50%	60%	70%	80%	90%	100%
n = 5										

0	0.44	0.44	0.47	0.47	0.47	0.61	0.66	0.66	0.66	0.67

0.5	0.50	0.50	0.50	0.50	0.50	0.58	0.62	0.62	0.62	0.62

1	0.53	0.53	0.49	0.49	0.49	0.55	0.58	0.58	0.58	0.58

2	0.60	0.60	0.48	0.48	0.48	0.50	0.52	0.52	0.52	0.52

5	0.65	0.65	0.40	0.40	0.40	0.40	0.42	0.42	0.42	0.43

10	0.65	0.65	0.34	0.34	0.34	0.34	0.36	0.36	0.36	0.38

n = 10										

0	0.50	0.35	0.45	0.54	0.61	0.66	0.67	0.69	0.70	0.70

0.5	0.56	0.40	0.47	0.55	0.60	0.63	0.65	0.65	0.66	0.66

1	0.60	0.42	0.48	0.54	0.58	0.60	0.61	0.61	0.61	0.61

2	0.68	0.46	0.47	0.51	0.53	0.55	0.55	0.56	0.56	0.56

5	0.77	0.43	0.37	0.38	0.39	0.41	0.42	0.43	0.44	0.44

10	0.83	0.36	0.29	0.31	0.33	0.34	0.35	0.36	0.37	0.37

n = 20										

0	0.40	0.38	0.50	0.58	0.65	0.69	0.71	0.72	0.72	0.72

0.5	0.46	0.41	0.51	0.58	0.63	0.65	0.67	0.67	0.67	0.67

1	0.48	0.44	0.51	0.56	0.60	0.61	0.61	0.62	0.62	0.62

2	0.55	0.46	0.49	0.53	0.55	0.56	0.57	0.57	0.57	0.57

5	0.60	0.41	0.40	0.42	0.44	0.46	0.46	0.47	0.47	0.47

10	0.56	0.33	0.30	0.32	0.33	0.36	0.37	0.38	0.39	0.39

n = 100										

0	0.32	0.42	0.56	0.64	0.69	0.72	0.74	0.74	0.74	0.74

0.5	0.36	0.45	0.57	0.63	0.67	0.69	0.70	0.70	0.70	0.69

1	0.39	0.46	0.56	0.60	0.63	0.65	0.65	0.65	0.65	0.65

2	0.44	0.48	0.56	0.59	0.61	0.61	0.61	0.61	0.61	0.61

5	0.47	0.42	0.46	0.47	0.48	0.49	0.50	0.50	0.50	0.51

10	0.45	0.33	0.34	0.36	0.38	0.39	0.40	0.40	0.41	0.41

The top early recognitions are represented as percentages.

### Statistical power: single ranking method

The null distribution helps determine a threshold above or below which a ranking method can be declared statistically significantly better than random ranking. However, for examining the specificity of the metrics, we also need to determine the probability (statistical power) that a ranking method is above or below this threshold when a ranking method is truly better than random ranking. We have adopted the simulation method outlined by Truchon and Bayley [16] for this purpose. Briefly, we assume a typical VS study with *N *total compounds in which there are *n *active compounds and *N *- *n *decoy compounds. The ranks of the *n *active compounds are drawn from an exponential distribution; *n *random numbers, {*u*_1_, *u*_2_, ..., *u*_*n*_}, from a uniform distribution, are first generated and then inverse transformed according to Eq (8). Their ranks are taken as the integer part of (*Nx*_*i *_+ 0.5),

(8)

as shown in Eq (9). *λ *is an important parameter that controls the earliness of response by which active compounds are ranked; a high *λ *value favors early recognition and *λ *= 0 corresponds to random ranking.

(9)

Assuming that *n *actives need to be simulated, Eq (10) shows how to calculate the probability that at least *m *actives are ranked in the top *z *of the list. For example, the probability that at least *m *= 1 active ranked in the top *z *= 50% of the list is 0.92 when *n *= 10 and *λ *= 5. The probabilities for different combinations of *m*, *λ*, and *z *are included in [Additional file 1].

(10)

**Figure 3:**
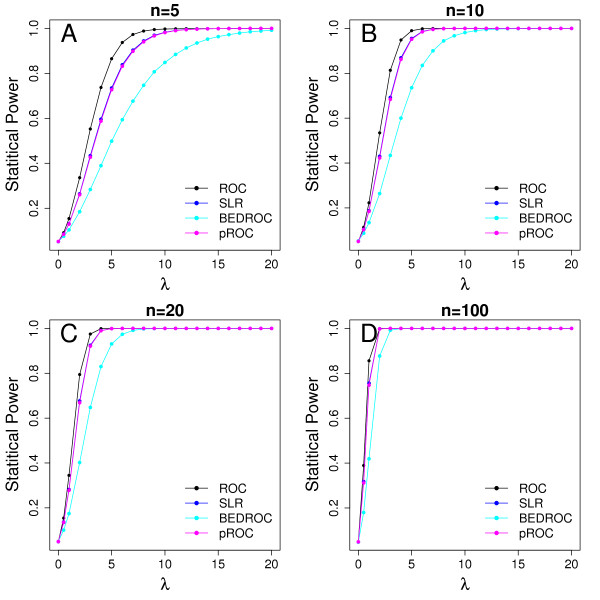
**Statistical power of different metrics for *λ *values from 0 to 20 for different number of actives (*n *= 5, 10, 20, 100) when comparing with random ranking**. Black line, AU-ROC; blue line, SLR; turquoise line, BEDROC; and red line, pROC.

Figure 3 shows the statistical power of SLR, AU-ROC, pROC, and BEDROC for different *λ *values, ranging from 0 to 20. AU-ROC is uniformly most powerful of among all metrics, SLR and pROC are tied as the second most powerful, and BEDROC is the least powerful metric. Because BEDROC overemphasize a very small proportion of actives, it is poorer than other metrics at detecting true early recognitions. A smaller *α *can increase statistical power but it should be carefully selected to make sure that early recognition is also addressed. The statistical power of all metrics approaches 1 when *λ *>15, confirming that all metrics are able to evaluate which ranking methods are better than random ranking.

When *λ *= 20, BEDROC returns to a value of 1/2 when *α *<< 1. The authors imply that 1/2 should be set as a threshold of early recognition "usefulness". The threshold at 0.01 type I error rate for BEDROC is 0.22 for *n *= 10, which is much lower than 1/2. For 1 million bootstraps under the null hypothesis, the maximum value for BEDROC value 0.354; thus, it is too stringent for BEDROC to use 1/2 as a threshold. Selecting an appropriate tuning parameter *α *for BEDROC is challenging; when *α *is too large, BEDROC considers only a small proportion of early recognized actives, and when *α *is too small, BEDROC becomes similar to AU-ROC.

### Statistical power: comparing two ranking methods

**Figure 4:**
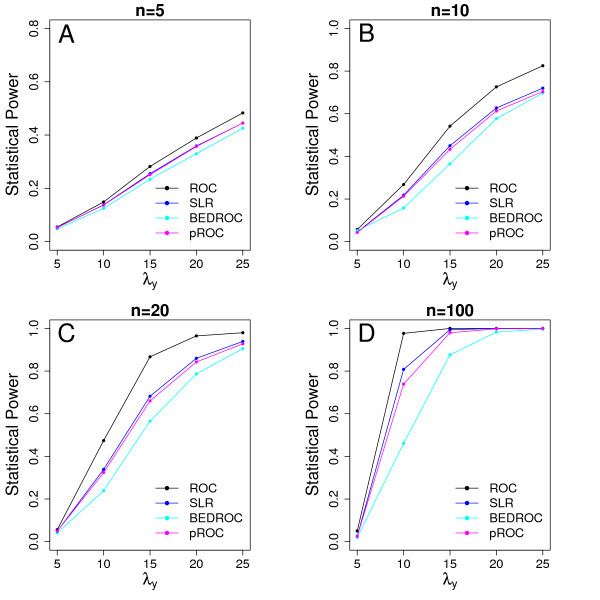
**Statistical power of different metrics at different *λ*_*y *_values for different number of actives (*n *= 5, 10, 20, 100) when comparing different ranking methods**. Black line, AU-ROC; blue line, SLR; turquoise line, BEDROC; and red line, pROC.

To assess the statistical power of comparing 2 ranking methods, we fix the tuning parameter of ranking method, *x*, with *λ*_*x *_= 5 and vary the tuning parameter *λ*_*y *_for the ranking method *y*. For a comparison, we randomly draw the ranks of actives from the pool of (*x*, *y*) and calculate the *p *value by the permutation test. Two ranking methods are considered significantly different if *p *value is less than 0.05. For each parameter setup, 10,000 comparisons are repeated. False-positive rates are evaluated when *λ*_*y *_= 5. As shown in [Additional file 2], false-positive rates are highest for AU-ROC and those for SLR, BEDROC, and pROC are comparable. Figure 4 shows the power of all metrics for different *λ*_*y *_values for different number of actives. When *λ*_*y *_> 5, AU-ROC is most powerful, followed by SLR; pROC is slightly less powerful than SLR but still much more powerful than BEDROC. For any fixed *λ*_*y *_= 5, the statistical power of all metrics increases as the number of actives is increased.

## Conclusion

Many metrics used in practice address the early recognition problem differently, but their performances can be adjusted by using different tuning parameters, *α *and *β*. However, an appropriate tuning parameter should be selected very carefully due to the "seesaw effect" observed in our simulation studies if a metric overemphasizes early recognition, it becomes too specific and only a very small proportion of actives have a play in the metric, and the metric loses statistical power to detect true early recognitions. This is seen in BEDROC at *α *= 20. Although the statistically significant ranking methods defined by BEDROC have smaller average ranks in the first 10–30% of the list than those defined by AU-ROC, SLR, and pROC, BEDROC is the least powerful of all metrics. On the other hand, AU-ROC gives equal importance to all actives and does not reward early recognition in particular, but it remains the most powerful test. A good tuning parameter should be based on the consideration of both statistical power and "early recognition". It is advisable to determine the null distribution by mathematical derivation, bootstrap samplings, or permutations before determining the threshold.

As a rule of thumb, 2 scores derived from 2 different compound lists cannot be compared directly. Our simulation studies- have shown that the null distribution of a metric changes with the number of actives. A threshold used in one study may not apply to other studies. Except AU-ROC, all other metrics do not have a probability meaning and cannot be interpreted in the probability language even if they are bounded by [0, 1].

The statistical guideline proposed in this paper can be used as a general procedure to make statistical comparisons in evaluating virtual screening studies. This guideline is applicable to any metric. Under null hypothesis, the theoretical distribution of SLR metric can be derived from Gamma distribution. The threshold for significance test is easily calculated without resorting to bootstrap simulations, which makes it practically useful in virtual screening studies and superior to other metrics.

## Authors' contributions

WZ drafted the manuscript, developed the SLR method and performed the data analysis. KH tested the method and proved its usefulness. SW, RL and JB provided substantial contributions to conception and design. All authors read and approved the final manuscript.

## Supplementary Material

Additional file 1 **The probability that active compounds are ranked top in the list**. The data provide the probabilities for a combination of different parameters. Click here for file

Additional file 2 **The statistical power to compare two ranking methods**. The data provide the statistical powers for different sample size and *λ*. Click here for file
