# Supplementary material for: A statistical framework to evaluate virtual screening
Source: BMC Bioinformatics. 2009 Jul 20;10:225. doi: 10.1186/1471-2105-10-225 (PMC2722655; doi:10.1186/1471-2105-10-225)
Supplement: Additional file 1 — The probability that active compounds are ranked top in the list. The data provide the probabilities for a combination of different parameters. [file 1471-2105-10-225-S1.doc]

**Additional files**

**Additional file 1- Probability that at least**
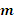
 **actives are ranked in the top**
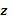
 **of the list for different**
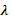


**Table S1:** Probability that at least
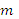
 actives are ranked in the top
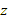
 of the list for different
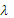
 values.

| *m* | λ =5 | 10 | 15 | 20 |
| --- | --- | --- | --- | --- |
|  |  | z=0.01 |  |  |
| 1 | 0.4 | 0.63 | 0.78 | 0.86 |
| 2 | 0.08 | 0.25 | 0.42 | 0.57 |
| 3 | 0.01 | 0.06 | 0.15 | 0.27 |
| 5 | 0 | 0 | 0.01 | 0.02 |
| 10 | 0 | 0 | 0 | 0 |
|  |  | z=0.05 |  |  |
| 1 | 0.92 | 0.99 | 1 | 1 |
| 2 | 0.69 | 0.95 | 0.99 | 1 |
| 3 | 0.39 | 0.82 | 0.96 | 0.99 |
| 5 | 0.05 | 0.35 | 0.69 | 0.88 |
| 10 | 0 | 0 | 0 | 0.01 |
|  |  | z=0.1 |  |  |
| 1 | 0.99 | 1 | 1 | 1 |
| 2 | 0.95 | 1 | 1 | 1 |
| 3 | 0.83 | 0.99 | 1 | 1 |
| 5 | 0.36 | 0.88 | 0.99 | 1 |
| 10 | 0 | 0.01 | 0.08 | 0.23 |

The number of actives is fixed at
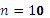
.
