# Supplementary material for: A statistical framework to evaluate virtual screening
Source: BMC Bioinformatics. 2009 Jul 20;10:225. doi: 10.1186/1471-2105-10-225 (PMC2722655; doi:10.1186/1471-2105-10-225)
Supplement: Additional file 2 — The statistical power to compare two ranking methods. The data provide the statistical powers for different sample size and λ. [file 1471-2105-10-225-S2.doc]

**Additional file 2- Statistical power to compare two ranking methods**

Table S2: Statistical power to compare 2 ranking methods.

|  | λ_y_ =5 | 10 | 15 | 20 | 25 |
| --- | --- | --- | --- | --- | --- |
|  |  | n=5 |  |  |  |
| AU-ROC | 0.055 | 0.149 | 0.282 | 0.389 | 0.483 |
| SLR | 0.053 | 0.138 | 0.255 | 0.359 | 0.445 |
| pROC | 0.053 | 0.137 | 0.251 | 0.357 | 0.445 |
| BEDROC | 0.048 | 0.125 | 0.234 | 0.33 | 0.426 |
|  |  | n=10 |  |  |  |
| AU-ROC | 0.057 | 0.268 | 0.542 | 0.726 | 0.825 |
| SLR | 0.045 | 0.218 | 0.45 | 0.627 | 0.72 |
| pROC | 0.043 | 0.213 | 0.433 | 0.613 | 0.704 |
| BEDROC | 0.052 | 0.158 | 0.365 | 0.577 | 0.697 |
|  |  | n=20 |  |  |  |
| AU-ROC | 0.056 | 0.474 | 0.867 | 0.965 | 0.98 |
| SLR | 0.049 | 0.339 | 0.682 | 0.86 | 0.939 |
| pROC | 0.049 | 0.324 | 0.66 | 0.843 | 0.928 |
| BEDROC | 0.041 | 0.239 | 0.565 | 0.787 | 0.905 |
|  |  | n=100 |  |  |  |
| AU-ROC | 0.05 | 0.977 | 1 | 1 | 1 |
| SLR | 0.026 | 0.808 | 0.994 | 1 | 1 |
| pROC | 0.025 | 0.739 | 0.98 | 0.998 | 0.999 |
| BEDROC | 0.02 | 0.461 | 0.876 | 0.983 | 0.997 |


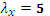
 and
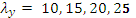
.
